# Using Population-Based Cancer Registration Data and Period Analysis to Accurately Assess and Predict 5-Year Relative Survival for Lung Cancer Patients in Eastern China

**DOI:** 10.3389/fonc.2021.661012

**Published:** 2021-05-11

**Authors:** Runhua Li, Min Zhang, Yongran Cheng, Xiyi Jiang, Huijuan Tang, Liangyou Wang, Tianhui Chen, Bicheng Chen

**Affiliations:** ^1^ Department of Cancer Prevention/Experimental Research Center, Cancer Hospital of the University of Chinese Academy of Sciences (Zhejiang Cancer Hospital), Institute of Basic Medicine and Cancer (IBMC), Chinese Academy of Sciences, Hangzhou, China; ^2^ School of Public Health, Hangzhou Medical College, Hangzhou, China; ^3^ Department of Non-Communicable Chronic Disease Control and Prevention, Taizhou Center for Disease Control and Prevention, Taizhou, China; ^4^ Key Laboratory of Diagnosis and Treatment of Severe Hepato-Pancreatic Diseases of Zhejiang Province, First Affiliated Hospital of Wenzhou Medical University, Wenzhou, China

**Keywords:** lung cancer, cancer registry, survival, period analysis, 5-year relative survival

## Abstract

**Background:**

The assessment of long-term survival of lung cancer patients based on data from population-based caner registries, using period analysis, was scarce in China. We aimed to accurately assess the long-term survival of lung cancer patients, and to predict the long-term survival in the future, using cancer registry data from Taizhou City, eastern China.

**Methods:**

Four cancer registries with high-quality data were selected. Patients diagnosed with lung cancer during 2004–2018 were included. The long-term survival was evaluated using period analysis, with further stratification by sex, age at diagnosis and region. Additionally, projected 5-year relative survival (RS) of lung cancer patients for 2019-2023 was evaluated, using model-based period analysis.

**Results:**

The 5-year RS of lung cancer patients diagnosed during 2014–2018 was 40.2% (31.5% for men and 56.2% for women). A moderate age gradient was observed for the period estimate, with the estimate decreasing from 50.5 to 26.5% in the age group of 15–44 years and ≥75 years, respectively. The 5-year RS of urban area was higher than that of rural area (52.3% vs. 38.9%). The overall projected 5-year RS of lung cancer patients was 52.7% for 2019–2023, with estimate of 43.0 and 73.2% for men and women, respectively. A moderate age gradient was also observed for the projected estimate. Moreover, estimate reached nearly 50% for rural and urban areas.

**Conclusion:**

Period analysis tended to provide the up-to-date and precise survival estimates for lung cancer patients, which is worth further application, and provides important evidence for prevention and intervention of lung cancer.

## Introduction

Lung cancer has been the leading morbidity and mortality in all cancers in China for many years, which has a rapidly growing trend in the past few decades and requires special attention. In China, 605,900 cases of lung cancer were diagnosed and 486,600 of lung cancer died in 2010 (1). Meanwhile, lung cancer was the leading cause of life-years lost in 2017 in China (2). Lung cancer is the most common cancer in Zhejiang Province, China and its morbidity and mortality also ranks first for all cancers. From 2010 to 2016, the crude incidence of lung cancer in Taizhou City, Zhejiang Province was 65.05 per 100,000 and the crude mortality rate reached 50.59 per 100,000 (3). Given that the morbidity and mortality of lung cancer have increased dramatically over the past few years and will continue to increase in the future in Taizhou City, the public health interventions and clinical management are necessary to reduce the burden and improve the survival of lung cancer. Therefore, timely assessment of long-term survival of lung cancer patients is essential for clinical management and public health interventions for lung cancer patients.

The relative survival (RS) is a key indicator of long-term survival, which reflects the level of cancer prevention and prognosis in a certain area (4, 5). There are some methods to calculate 5-year RS by cohort approach, complete analysis and period analysis for cancer patients. However, we choose period analysis and not other methods, because compared to traditional methods, the period analysis proposed by Brenner (6) is a new method and can evaluates the long-term survival more timely and accurately. It is considered as “gold standard” for the assessment of long-term survival of cancer patients using population-based cancer registry data and has been widely used in the western (6, 7), and has recently by our group in Chinese population (4). However, the period analysis cannot reflect the latest survival for cancer patients because the collection of cancer registry data is generally delayed for 1–3 years and a tradeoff between timeliness and precision of survival estimates has to be balanced. Therefore, Brenner and colleagues (8) further proposed the model-based period analysis in 2006, which is based on the generalized linear model, using the existing cancer registry data to predict subsequent survival in the upcoming period. The model-based period analysis can not only estimate the recent survival trend, but also predict the future survival rate using the cancer registry data (9).

Here, in our study, we aimed to provide the up-to-date and precise survival estimates for patients with lung cancer in 2014–2018, and to predict the long-term survival in 2019–2023 based on the model-based period analysis, using data from population-based cancer registry in Taizhou City, eastern China.

## Materials and Methods

### Data Source

The case data was retrieved from nine cancer registries of Taizhou City, during the period of 2004–2018. Data quality was assessed by the proportion of death certificate only (DCO) cases among all the cancer cases, with DCO% below 13% to be acceptable (8). Thus, data from four cancer registries (Luqiao, Yuhuan, Xianju and Wenling) were included for further analyses. Follow-up with respect to vital status was available until the end of December 31, 2018.

According to the International Classification of Diseases, 10th Revision (ICD-10), cases with the code of C33-C34 were identified as lung cancer patients. Therefore, an overall of 20,491 lung cancer patients were initially identified, and patients aged 15 years or older were included in this study. Among them, patients who were lost to follow-up (n = 1,172), unknown cases (n = 300), or missing at last follow-up (n = 2,907) were excluded. Eventually, overall 16,112 patients were retained for further analyses.

### Statistical Analysis

The 5-year RS of lung cancer patients was calculated, which is defined as the ratio of observed survival in the patient group divided by the expected survival of a comparable group from general population (4). The expected survival was derived from life-table for the four cities of Taizhou (Luqiao, Yuhuan, Xianju and Wenling) population stratified by age, region, and calendar year, using the Ederer II method (10).

Period analysis was firstly used to assess the 5-year RS of patients diagnosed in 2014–2018. The included cases were divided into two parts, one was the newly diagnosed patients in the interest period, and the other was the patients diagnosed before the interest period but still alive within the interest period. Specifically, the cases diagnosed from 2009 to 2018 who were still alive during 2014–2018 with the follow-up between 2014 and 2018 were included. Period analysis was used to deal with the left-censored data diagnosed before the interest period and the right-censored data still alive at the end of the interest period. Moreover, the method of period analysis was used to collect the data into a life table and calculate the 1-year RS *S_i_* at the *i* year of follow-up. The formula was as follows:

$$S_{i}=1-\frac{d_{i}}{n_{i}-c_{i}/2}$$

In this formula, *n_i_*represented the population at the beginning of the *i* year of follow-up, *d_i_* represented the number of deaths at the end of *i* year of follow-up, and *c_i_* represented the number of censored data in *i* year. The observed survival $\bar{S_{k}}$ of k-year was obtained by multiplying the k-year conditional one-year survival rate. The formula was as follows:

$$\bar{S_{k}}=\prod_{i=1}^{k}S_{i}$$

RS was the ratio of observed survival to expected survival. The formula was as follows:

$$R_{i}=\frac{\bar{S_{k}}}{S_{k}^{*}}$$

When calculated the 5-year RS, *k* was 5 in the above formula. Among this formula, $\bar{S_{k}}$ represented observed survival, and $S_{k}^{*}$ represented the expected survival. The point estimates of RS and their standard errors (SE) were calculated using the Greenwood method (4).

Next, the model-based period analysis was used to predict the 5-year RS of patients during the period of 2019–2023, with further stratification by sex, age at diagnosis and region. A generalized linear model (GLM) was established based on period analysis and complete cancer registries, estimating the survival of patients, analyzing the trend of survival changes, and predicting the future survival. For instance, using the latest follow-up data of cancer registries end of December 31, 2018, and data of three periods 2004–2008, 2009–2013, and 2014–2018, the survival of patients diagnosed in 2019–2023 will be predicted (**Table 1**).

**Table 1:** Schematic diagram of the model-based period analysis.

Diagnosis year	Follow-up year			
	2004–2008	2009–2013	2014–2018	2019–2023
**1999–2003**				
**2004–2008**				
**2009–2013**				
**2014–2018**				
**2019–2023**				

All statistical analyses were performed using the package ‘periodR’ of R version 3.13 (R Foundation for Statistical Computing, Vienna, Austria) (11).

## Results

### Basic Characteristics of the Patients

The basic characteristics of patients diagnosed in the 2004–2018 interval were shown in **Table 2**. Overall, 16,112 cases were included in the analyses, including 10,901 male and 5,211 female, respectively. The average age at diagnosis of patients was 65.9 years. Notably, patients diagnosed in the age group of 65–74 years accounted for the largest part, while the age group of 15–44 years accounted for the smallest proportion. Moreover, patients who lived in the urban and rural area were 2,489 and 13,623, respectively.

**Table 2:** Basic characteristics of the patients in 4 cancer registries of Taizhou City (2004–2018).

	Number of cases	Diagnosed interval		
		2004–2008	2009–2013	2014–2018
Gender				
Male	10,901	1,318	3,798	5,785
Female	5,211	464	1,533	3,214
Region				
Urban area	2,489	40	877	1,572
Rural area	13,623	1,742	4,454	7,427
Average age (years)	65.9	65.8	66.2	65.6
Age at diagnosis (years)				
<45	610	74	187	349
45–54	2,075	246	632	1,197
55–64	4,434	404	1,488	2,542
65–74	4,872	635	1,574	2,663
>74	4,121	423	1,450	2,248
Total	16,112	1,782	5,331	8,999

### Five-Year RS Estimate of the Patients by Period Analysis

As shown in **Table 3**, the 5-year RS of patients was 40.2% in the 2014–2018 interval, with the estimates of 31.5 and 56.2% for male and female patients, respectively. A moderate age gradient was observed for the period estimate, with the estimate decreasing from 50.5% in the age group of 15–44 years to 26.5% in the age group of ≥75 years. Moreover, prognosis varied by region, which showed that the 5-year RS of urban area (52.3%) was higher than that of rural area (38.9%).

**Table 3:** Survival of lung cancer patients in four cancer registries in Taizhou City from 2014 to 2018.

	Estimated value (%)	Standard error (SE)
Total	40.2	0.5
Gender		
Male	31.5	0.7
Female	56.2	0.8
Age at diagnosis (years)		
<45	50.5	1.6
45–54	42.9	1.5
55–64	36.0	0.9
65–74	32.1	0.8
>74	26.5	0.4
Region		
Urban area	52.3	1.1
Rural area	38.9	0.6

### Projected 5-Year RS Estimate of the Patients

**Figure 1:**
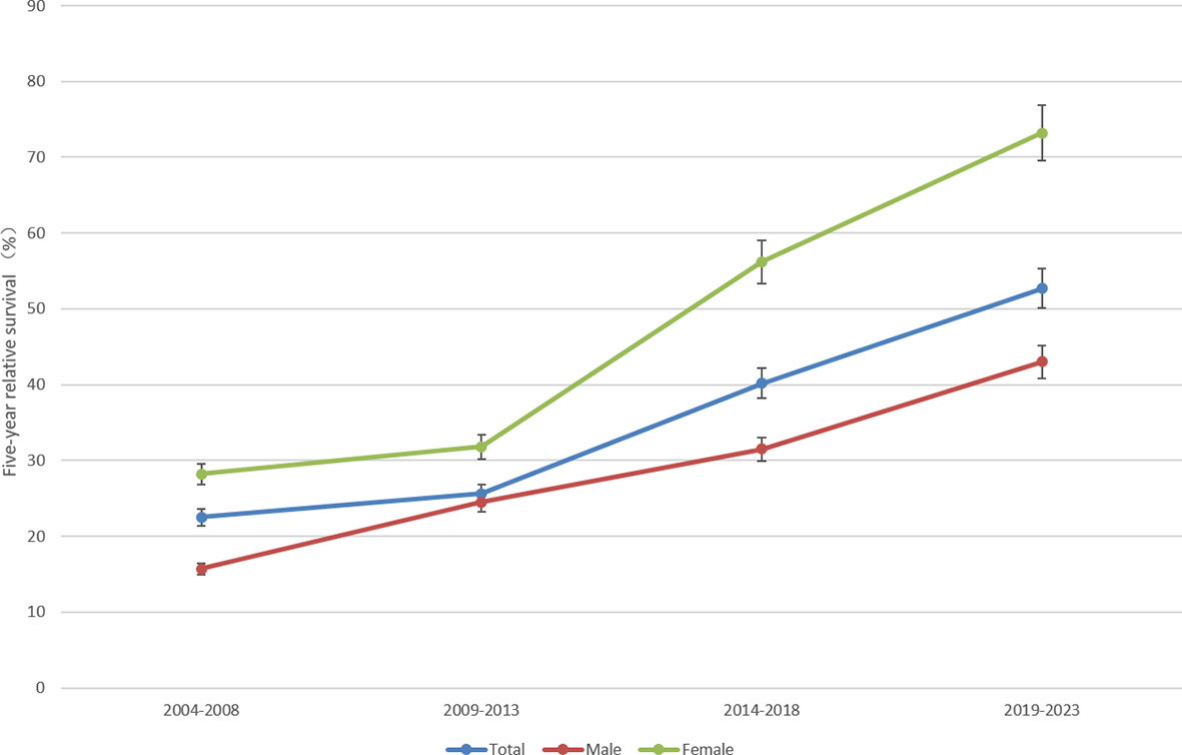
Five-year relative survival for total, male and female lung cancers in 2004–2008, 2009–2013, 2014–2018 and 2019–2023.

**Figure 2:**
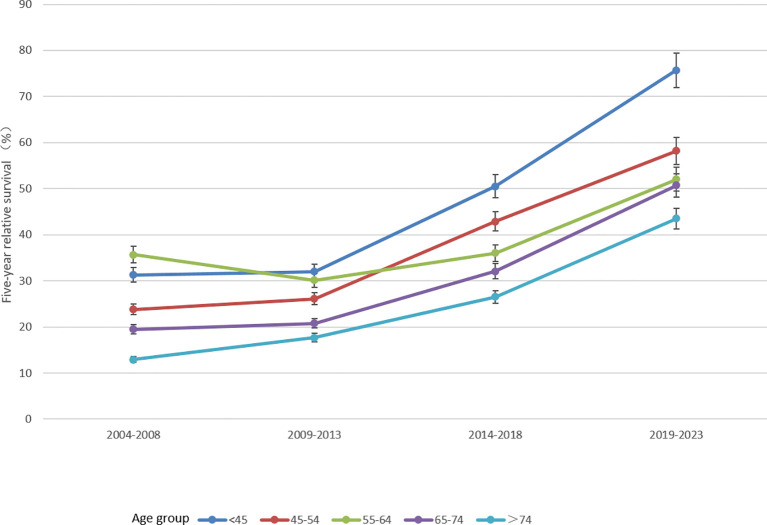
Five-year relative survival of lung cancers for different ages at diagnosis in 2004–2008, 2009–2013, 2014–2018 and 2019–2023.

**Figure 3:**
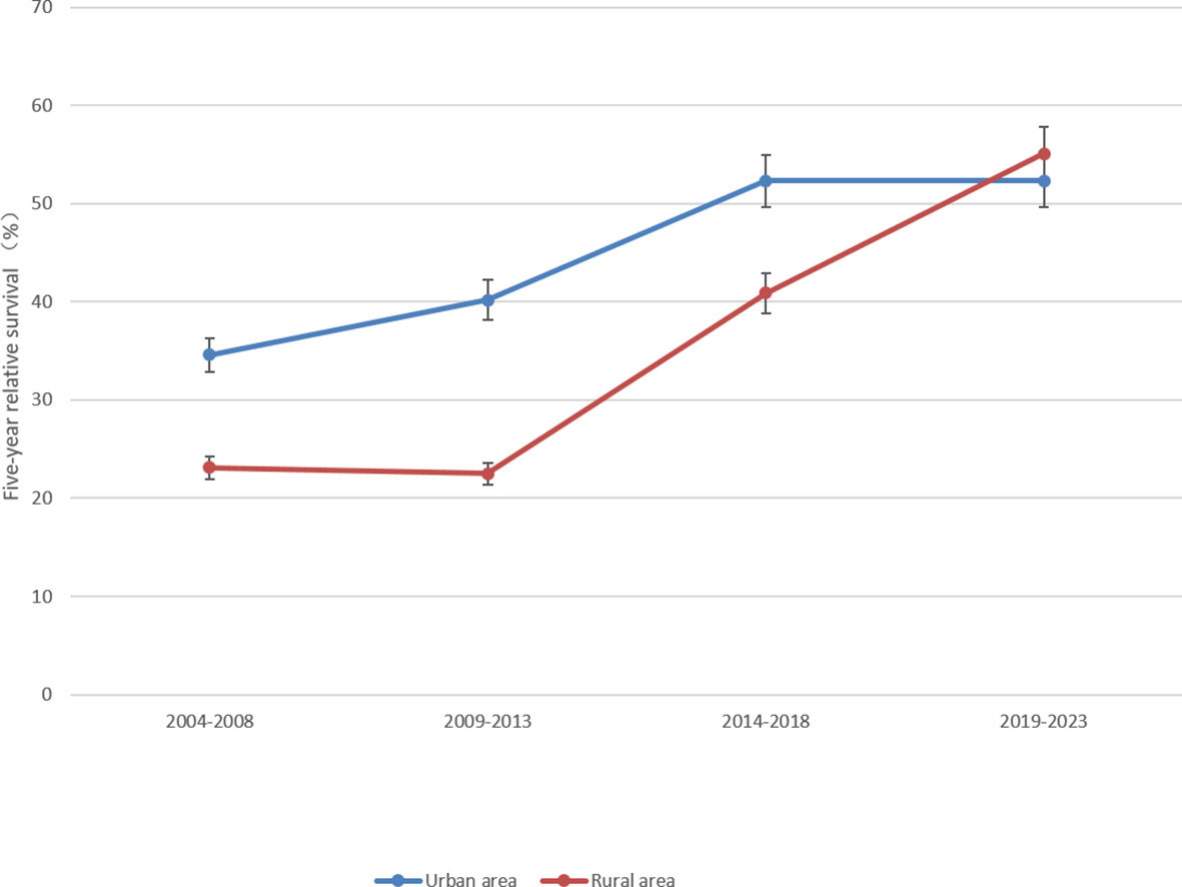
Five-year relative survival of lung cancers for urban and rural areas in 2004–2008, 2009–2013, 2014–2018 and 2019–2023.

The 5-year RS of patients during 2019–2023 was predicted to be 52.7% (**Table 4**). Overall, the 5-year RS showed an increasing tendency during the four periods (2004–2008, 2009–2013, 2014–2018 and 2019–2023). And, the estimates of 5-year RS of female patients were higher than those of male patients in all four periods. Compared with the period of 2009–2013, the gap in 5-year RS between male and female patients in the latter two periods was found to be gradually increasing. Moreover, except for the age group of 55–64 years which showed a downward trend during 2009–2013, the 5-year RS of patients at other diagnosed age showed an upward trend in all four periods. Compared with the period of 2014–2018, the 5-year RS of urban patients was predicted to decrease in 2019–2023, and the 5-year RS of rural patients would be higher than that of urban patients (**Figures 1**–**3**).

**Table 4:** Prediction of the survival of lung cancer patients in four cancer registries of Taizhou City from 2019 to 2023.

	Estimated value (%)
Total	52.7
Gender	
Male	43.0
Female	73.2
Age at diagnosis (years)	
<45	75.7
45–54	58.2
55–64	52.0
65–74	50.7
>74	43.5
Region	
Urban area	52.3
Rural area	55.1

## Discussion

The incidence of lung cancer is increasing in Taizhou, which brings numerous challenges to the economy in this region. The regional government has struggled to meet the needs of the healthcare for all population (12). Many studies have (5, 8, 13, 14) proved that period analysis provided more precise cancer survival estimates than complete and cohort methods, when using the large datasets. In our study, it demonstrated that although the number of lung cancer patients was increasing in the periods of 2004–2008, 2009–2013 and 2014–2018 (**Table 2**), the 5-year RS of patients were also improving in these three periods (**Figure 1**). This remarkable increase in long-term survival confirms a favorable trend of prognosis in lung cancer in Taizhou City, which was similar to the trend in other Asian countries like Japan (5) and Korea (14).

First, in China, since the medical system reform in 2009, the hospital community benefit movement has implemented effective strategies to focus on healthcare and expand medical insurance, in which the government has the primary responsibility to ensure that citizens, both in urban and rural areas, have access to affordable healthcare (15). Second, computed tomography (CT)-based lung cancer screening has been applied gradually in health screening in China. CT screening in China would prevent more than 0.72 million deaths and more than 5.8 million life-years lost, resulting in 6.30–6.58% and 1.97–2.79% mortality reduction in males and females with different guidelines, respectively (16). Without CT screening, 14.98 million deaths from lung cancer will occur between 2016 and 2050. Screening with low dose computed tomography (LDCT) has proven to be beneficial in reducing the mortality related to lung cancer, which is mainly based on early detection of cancer and timely initiation of treatment (17). Third, the treatment of lung cancer has improved and 20–30% of patients are eligible for curing with surgery (18). In addition to surgery, other therapies have also been developed for patients with primary or metastatic lung cancer recently, including radiation therapy, thoracoscopic segmentectomy and thermal ablation therapy. The 5-year overall survival and relapse free survival were 93.4 and 90.8% with lung segmentectomy under thoracoscopy, respectively (19). Even the survival of non-small cell lung cancer is also significantly longer with advanced treatment (20).

In order to solve the potential underestimation of long-term survival, the model-based period analysis used in our study tended to provide more accurate and timely long-term survival experiences for lung cancer patients. In our study, patients were divided into two parts: the new patients in the interest period and the patients diagnosed before the interest period but still alive within the interest period. Thus, the model-based period analysis adjusted data by left censoring data diagnosed before the interest period and right censoring data still alive at the end of the interest period. As far as we know, this is the first study to predict the RS of lung cancer patients based on data from population-based cancer registry in Taizhou City. The model-based period analysis eliminated the tradeoff between up-to-dateness and precision of period survival estimates (8, 9). Based on the model-based period analysis, a GLM was established to analyze the trend of survival changes and predict future survival for lung cancer patients. It was predicted that the 5-year RS of lung cancer patients in Taizhou City would be 52.7% in 2019–2023 (**Table 4**), compared with 40.2% in 2014–2018 (**Table 3**). Meanwhile, the next 5-year survival estimates for lung cancer in this study were considered to be more reliable because the population-based data was more than 10 years. In addition to the reasons mentioned above, the trend of increasing survival in the near future for lung cancer is related to the well-educated population in Taizhou City who are more relatively aware of the importance of being physically fit than other regions (21). Moreover, Taizhou City located in eastern China, which has fast-growing economy and perfect medical insurance system (4).

The survival of lung cancer patients was further stratified by age at diagnosis, sex and region in this study. Age at diagnosis between 65 and 74 years had the largest number of lung cancer patients and the average age at diagnosis was 65.9 years (**Table 2**), similar to other studies (14, 22). In addition, the 5-year RS decreased with the increase of age at diagnosis (**Tables 3**, **4**), suggesting that the earlier the discovery, the higher the survival. Notably, the 5-year RS of male patients were lower than that of female patients in all four periods and the gap between male and female gradually increased. Many studies have certificated that the incidence and mortality rates of lung cancer in males were higher than that in females both in China (1, 3) and abroad (23), and the 5-year RS was lower among males (24). Smoking is the major cause of lung cancer. More than half of males in China are now smokers and an unprecedented number of deaths from smoking will occur as China’s population ages (16). There is a more favorable prognosis of lung cancer for never smokers compared to smokers (25). Exposure to secondhand tobacco in house (23) and environmental pollution (26) are the main risk factors that contribute to non-smoking women with lung cancer. Moreover, current smokers diagnosed with lung cancer are younger than former smokers, and increasing proportions of smokers were among older populations (27). Therefore, smoking may be the reason attributed to the differences in survival of lung cancer between men and women. So comprehensive smoking cessation at the earliest age and decreasing secondhand tobacco exposure may reduce the development of lung cancer and improve the survival of lung cancer, especially for middle-aged and old male patients.

Most surprisingly, in our study we found that the number of lung cancer patients who was lived in rural area was about five times in urban area in 2009–2013 and 2014–2018. The 5-year RS of urban and rural areas were 52.3 and 38.9% in 2014–2018, respectively. But in 2019–2023, the 5-year RS in the rural area reached 55.1%, which was higher than that of urban area (52.3%). The rural area is an independent risk factor for decreased survival in lung cancer, especially for non-small cell lung cancer, which is related to the lower Medicaid expansion (28), less CT screening (29) and less advanced treatment (30). Meanwhile, the rural population has higher rates of smoking compared with the urban population (26). Therefore, expanding medical insurance (15), quitting smoking and strengthening screening and treatment in the rural areas, especially for those who are low-income, may greatly improve lung cancer survival in the future. The survival of the rural area will be greatly improved in our prediction results, which means that expanded healthcare and effective interventions could effectively improve survival for the rural population in Taizhou City. It is worth noting that the increasing implementation of chest CT screening is expected to find out the high-risk populations (31), and strengthening the standardized treatment of lung cancer at all levels in hospitals will significantly increase the 5-year RS of lung cancer patients in Zhejiang Province.

This study has some limitations. First, data calculating the long-term survival was only from four cancer registries in Taizhou City, and thus the number of lung cancer patients that can be estimated is limited. Considering the increasing trend in the incidence of lung cancer, estimates of cancer survival might require larger data to obtain a stable estimation and include more population-based cancer registration data in Zhejiang Province. Second, there was no other method used in this study to calculate long-term survival compared to relative survival, such as cause-specific survival (22). Third, further analyses stratified by other variables, such as smoking status, histological type, and treatment, were not conducted due to the lack of data. Therefore, further investigations which included more informative variables are warranted. In addition, period analysis also has disadvantages. Survival estimates required a trade-off between timeliness and accuracy. The period covered by the period analysis was relatively short and was the most recent years, so its timeliness was guaranteed. However, in the case of small population coverage and few cases in the cancer registry, the random variation increased due to the reduction in the number of patients at risk (8). Despite these limitations, data from nine cancer registries of Taizhou City was reasonable with high quality, however, according to the rule of DCO, data from four cancer registries was further analyzed with an acceptable proportion of death certificate less than 13%. In addition, lung cancer patients were selected from the database according to the ICD-10 coded C33–C34, deleting follow-up lost, unknown and missing cases. Therefore, long-term survival calculated in this study is reliable and rigorous.

## Conclusions

In the four 5-year intervals from 2004 to 2023, the overall 5-year RS of lung cancer is gradually increasing. It is predicted that the age gradient will continue to maintain between 2019 and 2023. Therefore, period analysis tended to provide the most timely and accurate long-term survival for lung cancer patients using population-based cancer registry.

## Data Availability Statement

The raw data supporting the conclusions of this article will be made available by the authors, without undue reservation.

## Author Contributions

TC was responsible for the study concept and design. TC, BC, and XJ obtained funding. LW and TC acquired data. YC and XJ analyzed and interpreted data. RL, MZ, YC, XJ, BC, LW, and TC drafted the manuscript, and all authors revised it for important intellectual content. TC is the guarantor of this work. All authors contributed to the article and approved the submitted version.

## Funding

This work was supported by grants from National Key Research-Development Program of China (2017YFC0908200 and 2019YFE0198800), Joint Key Program of Zhejiang Province-Ministry of Health (WKJ-ZJ-1714), Key Research-Development Program of Zhejiang Province (2017C03013), Youth Foundation of Zhejiang Academy of Medical Sciences (2019Y007), Health and Family Planning Commission of Zhejiang Province (2021KY131) and Start-up Funds for Recruited Talents in Zhejiang Cancer Hospital. The funding agencies had no role in the design and conduct of the study; collection, management, analysis, and interpretation of the data; preparation, review, or approval of the manuscript; and decision to submit the manuscript for publication.

## Conflict of Interest

The authors declare that the research was conducted in the absence of any commercial or financial relationships that could be construed as a potential conflict of interest.
